# A multi-task deep learning model for EGFR genotyping prediction and GTV segmentation of brain metastasis

**DOI:** 10.1186/s12967-023-04681-8

**Published:** 2023-11-07

**Authors:** Zichun Zhou, Min Wang, Rubin Zhao, Yan Shao, Ligang Xing, Qingtao Qiu, Yong Yin

**Affiliations:** 1 Department of Radiation Oncology and Physics, Shandong Cancer Hospital and Institute, Shandong First Medical University and Shandong Academy of Medical Sciences, 440 Jiyan Road, Jinan, 250117 Shandong China; 2 Department of Radiation Oncology, Shandong Cancer Hospital and Institute, Shandong First Medical University and Shandong Academy of Medical Sciences, 440 Jiyan Road, Jinan, 250117 Shandong China; 3 Department of Radiation Oncology and Technology, Linyi People’s Hospital, 27 Jiefang Road, Linyi, 276003 Shandong China; 4 Shanghai Chest Hospital, School of Medicine, Shanghai Jiaotong University, 241 Huaihai West Road, Shanghai, 200030 China

**Keywords:** Lung adenocarcinoma, Brain metastasis, EGFR, GTV, Deep learning, Multi-task model

## Abstract

**Background:**

The precise prediction of epidermal growth factor receptor (EGFR) mutation status and gross tumor volume (GTV) segmentation are crucial goals in computer-aided lung adenocarcinoma brain metastasis diagnosis. However, these two tasks present continuous difficulties due to the nonuniform intensity distributions, ambiguous boundaries, and variable shapes of brain metastasis (BM) in MR images.The existing approaches for tackling these challenges mainly rely on single-task algorithms, which overlook the interdependence between these two tasks.

**Methods:**

To comprehensively address these challenges, we propose a multi-task deep learning model that simultaneously enables GTV segmentation and EGFR subtype classification. Specifically, a multi-scale self-attention encoder that consists of a convolutional self-attention module is designed to extract the shared spatial and global information for a GTV segmentation decoder and an EGFR genotype classifier. Then, a hybrid CNN-Transformer classifier consisting of a convolutional block and a Transformer block is designed to combine the global and local information. Furthermore, the task correlation and heterogeneity issues are solved with a multi-task loss function, aiming to balance the above two tasks by incorporating segmentation and classification loss functions with learnable weights.

**Results:**

The experimental results demonstrate that our proposed model achieves excellent performance, surpassing that of single-task learning approaches. Our proposed model achieves a mean Dice score of 0.89 for GTV segmentation and an EGFR genotyping accuracy of 0.88 on an internal testing set, and attains an accuracy of 0.81 in the EGFR genotype prediction task and an average Dice score of 0.85 in the GTV segmentation task on the external testing set. This shows that our proposed method has outstanding performance and generalization.

**Conclusion:**

With the introduction of an efficient feature extraction module, a hybrid CNN-Transformer classifier, and a multi-task loss function, the proposed multi-task deep learning network significantly enhances the performance achieved in both GTV segmentation and EGFR genotyping tasks. Thus, the model can serve as a noninvasive tool for facilitating clinical treatment.

## Introduction

Lung cancer (LC) is one of the most common malignancies and remains the leading cause of cancer-related death [1]. Lung adenocarcinoma (LADC) is by far the most common subtype, accounting for 50% of all LC cases, and its incidence is on the rise [2]. As a result of late diagnosis and high heterogeneity, many LADC patients may develop brain metastasis (BM). This serious complication may cause issues ranging from mild headaches and cognitive impairments to seizures, focal neurological deficits, and even comas. Furthermore, patients who have untreated BM may die within one to three months [3, 4]. Thus, timely and effective treatment could have a positive impact on the physical conditions and quality of life of such patients. During the treatment process, brain radiotherapy combined with targeted drug therapy can effectively enhance the resulting therapeutic effect [5, 6].

Clinical studies have found that the epidermal growth factor receptor (EGFR) genotype (wild-type or mutation) has a significant impact on the treatment and prognosis of LADC BM patients because EGFR tyrosine kinase inhibitors (TKIs) can significantly improve LADC patients’ progression-free survival odds [7, 8]. Additionally, third-generation EGFR-TKIs were proven to have high blood-brain barrier permeability [9], which is necessary for LADC BM treatment. Some studies revealed that BM patients treated simultaneously with stereotactic radiosurgery (SRS) and EGFR-TKIs may have prolonged overall survival [10–12]. This makes EGFR mutation genotype sequencing as important as medical brain image testing. However, identifying EGFR genotypes through a biopsy is an invasive and costly procedure. In addition, magnetic resonance imaging (MRI) offers better soft tissue contrast than other imaging modalities, resulting in clearer visualizations of tumor characteristics [13]. In recent years, multiple studies have proven that BM MRI features are associated with EGFR genotype information [14–16]. Wang et al. [14] developed a radiomics model to predict EGFR mutation statuses for BM patients, and the model attained a classification accuracy of 0.845 on an independent testing dataset. Ye et al. [15] used a radiomics model to predict EGFR mutation statuses from T2-Flair and T1-weighted construct-enhanced (T1-CE) images and abtained effective prediction results (0.95 accuracy versus 0.867). While radiomics can improve the accuracy and efficiency of EGFR genotype prediction, it also has some structural limitations such as overfitting and underfitting caused by inappropriate feature dimensions and high model complexity. Haim et al. [16] used a convolutional neural network (CNN) to predict EGFR mutation statuses, achieving a mean accuracy of 0.898, a sensitivity of 0.687, and a specificity of 0.977. This research demonstrated the great potential of deep learning models.

Radiation therapy is one of the main treatments for BM patients to prolong their survival and improve their quality of life [17]. Patients with limited BMs can be treated with SRS instead of whole-brain radiation therapy [17, 18]. SRS utilizes three-dimensional image guidance and high conformal treatment planning to deliver a high radiation dose to the tumor area and a small dose to the adjacent normal tissue, achieving lasting BM control with minor side effects [19]. Before planning an SRS treatment, it is imperative to meticulously delineate the gross target volume (GTV). Although unified principles and consensuses are available for the delineation of the GTV, the delineation process is still mainly based on the radiation oncologist’s experience [20]. This process requires a high degree of concentration and is time-consuming. Currently, the applications of artificial intelligence (AI) are increasing in the field of radiotherapy for malignant brain tumors [21]. Li et al. [22] developed a two-stage deep learning model for the automatic detection and segmentation of BMs in MR images, yielding a segmentation Dice score of 0.81 and a detection precision of 0.56. Yu et al. [23] proposed a novel deep learning model to incorporate object-level detection into pixel-wise segmentation to simultaneously localize BMs and delineate contours, achieving a detection sensitivity of 0.91 and a detection precision of 0.77 on a small BM group and a segmentation Dice score of 0.86 on a large BM group. Hsu et al. [24] used 3D V-Net to segment BMs on MR and CT images. The experimental results showed that 3D V-Net achieved an overall sensitivity of 0.9 and a segmentation Dice score of 0.76. These existing works mainly focused on automatic BM detection, sacrificing detection precision and producing many false-positive results. Thus, these methods are more suitable for early brain metastasis detection than for auxiliary delineation after the diagnosis process.

In addition, deep learning networks can achieve multi-task collaboration using features extracted from MR image slices containing BM, which emphasizes the connections between the BM region and EGFR mutation statuses. Therefore, utilizing the BM GTV segmentation task as a constraint, the encoder of a deep learning network can directly focus on BM regions, thereby enhancing the accuracy of EGFR mutation status prediction in the network.

In this study, we employ MR image slices that have been diagnosed as having brain metastases by radiologists as the input images and propose a multi-task deep learning network to achieve EGFR mutation status prediction under the constraint of GTV segmentation. First, a multi-scale self-attention encoder is used to extract feature maps for the GTV segmentation decoder and EGFR status classifier. Second, we introduce a hybrid CNN-Transformer classifier to combine the feature maps and predict the EGFR status. Third, a multi-task loss function is employed to balance the performance attained in the segmentation and classification tasks. Our proposed model achieves state-of-the-art performance in a comparison with several existing single-task deep learning models.

## Materials and methods

### Network architecture design

**Fig. 1:**
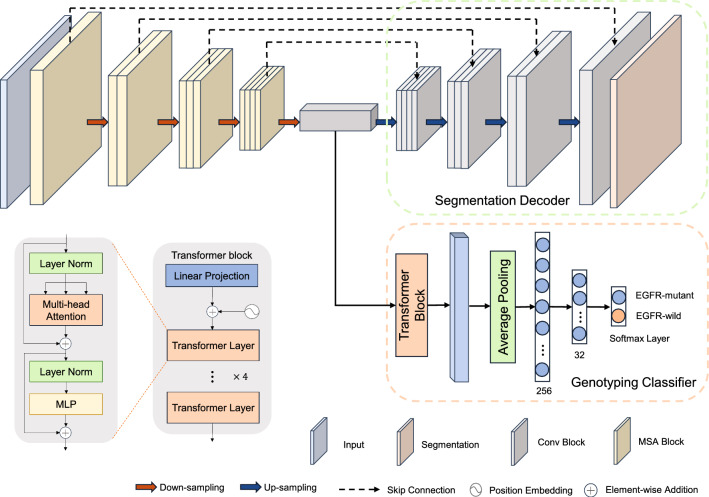
The architecture of MTSA-Net. The structure of the Transformer block is depicted in the lower-left corner. MLP denotes a multilayer perceptron. The MSA block is a multi-scale attention block

As illustrated in Fig. 1, the main architecture of the multi-task self-attention network (MTSA-Net), including a backbone, an EGFR genotyping classifier, and a GTV segmentation decoder, is implemented by using PyTorch (https://pytorch.io) [25]. MTSA-Net is a deep learning model designed for multi-task learning, where two tasks share a common feature space. Figure 1 shows that the output module of MTSA-Net is bifurcated into two components: a segmentation decoder and a genotyping classifier. The segmentation decoder is responsible for generating BM GTV prediction results that maintain the same size as the input image. These predictions effectively segment the image into the BM region and the background region. The genotyping classifier takes the high-dimensional feature map from the shared feature space as its input. It utilizes a Transformer module for feature fusion, followed by multi-layer dimensionality reduction operations. Finally, the classifier employs a softmax layer to predict the probability of the patient’s EGFR mutation status. Simultaneously, based on the multi-task approach, the loss function of the MTSA-Net encompasses both two tasks’ loss function. The GTV segmentation loss function serves to guide the network, directing attention to the BM region and enhancing the EGFR genotyping accuracy. The detailed descriptions of the specific implementation of each structure and loss function are provided below.

#### Backbone

**Fig. 2:**
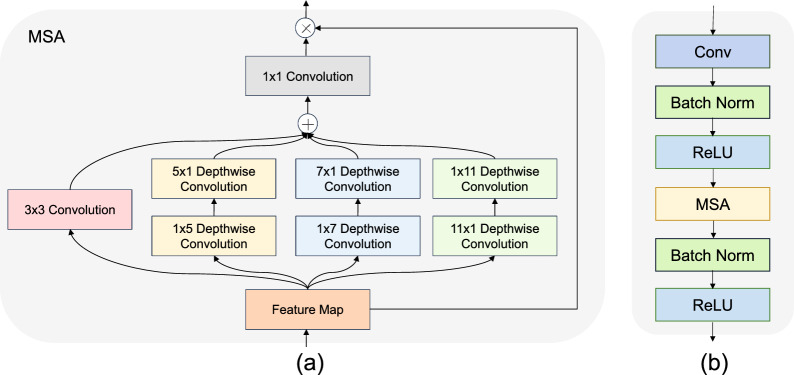
**(a)** represents the multi-scale attention layer and **(b)** is the multi-scale attention block

As shown in Fig. 1, the architecture of the encoder consists of multi-scale attention (MSA) blocks and downsampling operations. Each MSA block employs a series of convolutional blocks and MSA modules, inspired by [26], to extract semantic features from the input with a 2D spatial resolution of $384 \times 384$. As depicted in Fig. 2 (a), we adopt a pyramid structure and depth-wise convolution in the MSA module to obtain a multi-scale structure and a self-attention mechanism. The MSA module contains two parts: a $3 \times 3$ convolution is used to obtain local information, and multi-branch depth-wise convolutions are used to capture the multi-scale context of the input feature map. In the depth-wise convolution operations, we only need a pair of $k \times 1$ and $1 \times k$ convolutions [27]. The output of the $1 \times 1$ convolution after performing element-wise addition is employed as an attention weight to reweight the input feature map within the MSA module. The formula is as follows:

1 $$\begin{aligned} \begin{array}{c} Att = Con{v_{1 \times 1}}\left( {Con{v_{3 \times 3}}\left( F \right) + \sum \limits _{i = 1}^3 {Branc{h_i}\left( {DWConv\left( F \right) } \right) } } \right) \\ Out = Att \otimes F \end{array} \end{aligned}$$

where *F*, *Att*, and *Out* represent the input feature map, attention map and output feature map, respectively. $\otimes$ is the element-wise matrix multiplication operation. *DWConv* denotes depth-wise convolution. $Branc{h_i},i \in \left\{ {0,1,2,3} \right\}$ denotes the ith branch in Fig. 2 (a) from left to right. As illustrated in [28], the kernel sizes of the different branches are set to 5, 7, and 11.

As shown in Fig. 2 (b), the MSA block contains a $3 \times 3$ convolution, batch normalization, a rectified linear unit (ReLU) [29] activation function, and a MSA module.

#### Classifier for EGFR genotyping

**Fig. 3:**
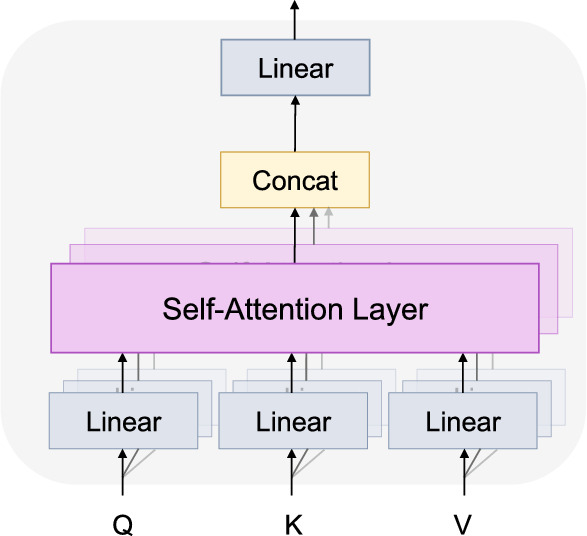
The architecture of the multi-head attention mechanism

**Fig. 4:**
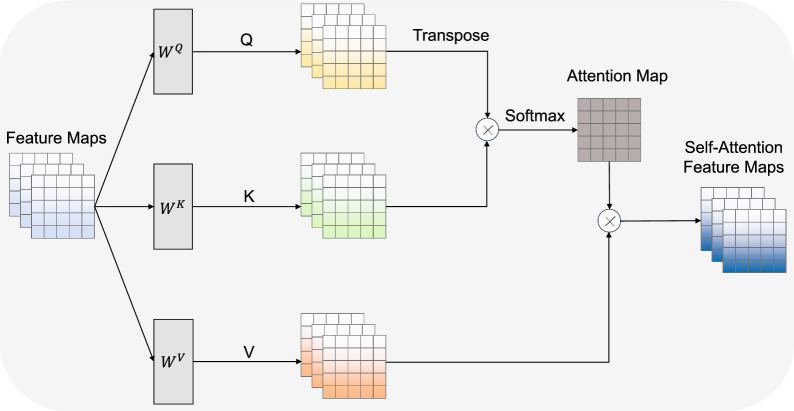
The architecture of the self-attention layer

To refine the feature map output by the MSA block and improve the combination of global and local information, we employ multi-head self-attention mechanisms in the classifier by using transformer blocks. The decoder consists of transformer blocks, convolutional blocks, a multilayer perceptron (MLP), and a softmax layer. Figure 1 shows the transformer block architecture containing multi-head attention and a feed-forward network. The multi-head attention is an essential part of the transformer layer, as it plays a crucial role in enabling the model to capture spatial relationships and dependencies across different regions within the input feature map. As shown in Fig. 3, the multi-head attention mechanism consists of multiple self-attention layers. The functionality of multi-head attention involves dividing the feature map of the transformer layer into multiple separate segments. Each segment is then subjected to self-attention operations performed independently and in parallel. The self-attention layer is illustrated in Fig. 4, and is computed as follows:

2 $$\begin{aligned} \begin{array}{l} Q = X \cdot {W^Q}\\ K = X \cdot {W^K}\\ V = X \cdot {W^V} \end{array} \end{aligned}$$

3 $$\begin{aligned} Attention(Q,K,V) = Softmax(\frac{{Q{K^T}}}{{\sqrt{d} }})V \end{aligned}$$

where *X* denotes the feature maps; *Q*, *K* and *V* denote the query, key and value of the input feature maps, respectively; $W^Q$, $W^K$ and $W^V$ are the trainable transformation matrices corresponding to the query, key and value, respectively; and *d* represents the dimensionality of the key vector.

In the multi-head attention mechanism, the outputs of the self-attention layers are concatenated and passed through a linear projection layer for further processing. This step can be computed as follows:

4 $$\begin{aligned} \begin{array}{c} Multi\,\,Head\left( {Q,K,V} \right) = Concat\left( {hea{d_1}, \cdot \cdot \cdot ,hea{d_h}} \right) {W^O}\\ hea{d_i} = Attention\left( {{Q_i},{K_i},{V_i}} \right) \end{array} \end{aligned}$$

where *Concat* is the concatenation operation layer; $W^O$ is the linear projection layer; $hea{d_i}$ is the *i*th self-attention head in the multi-head attention mechanism; and $Q_i$, $K_i$, $V_i$ represent the query, key and value in the *i*th self-attention head, respectively.

In our proposed model, the transformer block divides the input $24 \times 24$ feature map into 16 patches and reshapes each patch into a $6^2$ length sequence as a token input. Global average pooling with a kernel size of 4 is used to flatten the feature map output by the transformer block into a one-dimensional vector. An MLP is used to reduce the dimensionality of the feature map. Then, we employ a softmax layer to quantify the confidence index of the EGFR subtype as the final score.

#### Decoder for GTV segmentation

To obtain GTV segmentation results from T1-CE MR images, we design a 2D CNN decoder to implement voxel-level segmentation. The decoder comprises multiple cascaded convolutional blocks and skip connections, which merge the high-level and low-level feature maps and output the final segmentation result. Each convolutional block consists of a transposed convolution with a stride of 2 and a convolutional block. Transposed convolution is employed to achieve the upsampling operation. After completing the upsampling step, the feature map goes through a convolutional block consists of a $3 \times 3$ convolution, batch normalization, and a ReLU activation function. To predict precise GTV segmentation results with richer spatial details, skip connections are used to fuse the high-level feature maps and their low-level counterparts. Finally, a $1 \times 1$ convolution is applied to adjust the number of channels and produce the ultimate GTV segmentation result.

### Multi-task loss function

Since most of the regions in MR images do not contain BMs, segmentation bias may arise due to the imbalance between the foreground and background. To solve this problem, the Dice coefficient-based loss function and focal loss function [30] are employed as the segmentation losses for the GTV segmentation process, and they are defined as follows:

5 $$\begin{aligned} {L_{GTV}} = {L_{Dice}} + {\beta L_{Focal}} \end{aligned}$$

where ${L_{Dice}}$ is the Dice coefficient-based loss function, and ${L_{Focal}}$ is the focal loss function. Since there is an enormous difference between the numerical values of ${L_{Dice}}$ and ${L_{Focal}}$, $\beta = \frac{1}{{25,000}}$ is used to proportionally scale ${L_{Focal}}$ to reach the same level as ${L_{Dice}}$.

The Dice coefficient highlights the shape similarity between the predicted results and the ground truth, and is defined as:

6 $$\begin{aligned} {L_{Dice}} = 1 - Dice = 1- \frac{{2\left| {X \cap Y} \right| }}{{\left| X \right| + \left| Y \right| }} = 1 - \frac{{2\sum \limits _{i = 1}^S {{p_i}{y_i}} }}{{\sum \limits _{i = 1}^S {({p_i} + {y_i}) + \varepsilon } }} \end{aligned}$$

where S denotes the number of pixels, $p_i$ is the predicted probability of the proposed segmentation network, $y_i$ is the ground truth label, and $\varepsilon$ is a smoothing factor for avoiding division by 0.

The focal loss is a variant of the cross-entropy loss function that aims to balance the positive and negative samples. By adjusting the balancing parameter $\alpha$ and tuning parameter $\gamma$, the loss can better focus on hard samples and avoid the overwhelming the gradient with the larger number of false negatives. As mentioned in [30], it is preferable to set $\gamma$ to 2 in order to facilitate convergence while increasing the weights of hard samples. The loss function is defined as:

7 $$\begin{aligned} {L_{Focal}} = - \sum \limits _{i = 1}^S {{\alpha _i}{{(1 - {p_i})}^\gamma }\log ({p_i})} \end{aligned}$$

where S denotes the number of pixels and $p_i$ is the probability that the prediction is positive. $\alpha _i$ is the balancing parameter, and $\gamma$ is the tuning parameter. After performing parameter sweeping, $\alpha _i$ is set to 0.8, and $\gamma$ is set to 2.

The cross-entropy loss is used as the classification loss function to measure the difference between the model’s prediction and the ground truth. The classification loss function is defined as:

8 $$\begin{aligned} {L_{EGFR}} = - \sum \limits _{n = 1}^K {{p_n}} \log ({q_n}) \end{aligned}$$

where *K* represents the number of samples. $p_n$ and $q_n$ denote the ground truth of EGFR mutation status and the predicted result for sample *n*, respectively.

In our study, we utilize a multi-task loss function that optimizes the model by combining the segmentation loss and classification loss. The task weights setting are crucial for training multi-task models. To better adjust the task weights , we employ an uncertainty-based method that automatically weights the GTV segmentation and EGFR genotyping losses [31]. The multi-task loss is defined as follows:

9 $$\begin{aligned} {L_{\mathrm{{joint}}}} = \frac{1}{{2\sigma _{GTV}^2}}{L_{GTV}} + \frac{1}{{2\sigma _{EGFR}^2}}{L_{EGFR}} + \log {\sigma _{GTV}}{\sigma _{EGFR}} \end{aligned}$$

where $\sigma _{GTV}$ and $\sigma _{EGFR}$ represent the learnable parameters for multi-task learning. They are initially set to two tensors with values of 1.

## Experiment and analysis

### Datasets

The internal dataset, which includes T1-CE MR images and EGFR statuses of LADC BM patients, was derived from Shandong Cancer Hospital and Institute. A total of 188 patients diagnosed with LADC BM from August 2018 to October 2021 were enrolled. Institutional review board approval was received (SDTHEC2023007020), and the requirement to obtain written informed consent was waived due to the nature of the retrospective study. All MR images related to this study were anonymized. All patients in the internal dataset were imaged using a GE Discovery MR 750W scanner with six head coil channels in the same posture. The patients’ characteristics are summarized in Table 1. All patients were ordered according to the times at which they were diagnosed at Shandong Cancer Hospital and Institute. We removed 33 patients diagnosed after a cutoff date of May 1st, 2021 from the whole dataset into the internal testing set, and the rest patients were divided into the training group. Subsequently, a validation set comprising 10 patients was randomly selected from the training group to determine the optimal model weights during the training process. Therefore, this approach produced a testing environment closer to the real clinical situation, which was more consistent with the grouping specification in the TRIPOD statement [32].

**Table 1:** The statistics of the training group and internal testing set used in this study

		Training group (n = 155)	Internal testing set (n = 33)	P-value
Age				0.425
Median(range)		57 (46–78)	56 (48–72)	
Sex				0.276
Male		90 (58.06%)	20 (60.61%)	
Female		65 (41.94%)	13 (39.39%)	
Smoking				0.135
Yes		61 (39.35%)	10 (30.30%)	
No		94 (60.65%)	23 (69.70%)	
EGFR Status				0.149
Mutant		83 (53.55%)	19 (57.58%)	
Wild		72 (46.45%)	14 (42.42%)	

The external testing set was derived from two additional public hospitals. Twenty-two patients diagnosed with LADC BM at Linyi People’s Hospital and 16 patients diagnosed with LADC BM at Shanghai Chest Hospital were enrolled. All the cases included in the external testing set possessed precise T1-CE MR images and EGFR mutation statuses. The T1-CE MR images were obtained using Siemens MAGNETOM Lumina, Siemens MAGNETOM Prisma, Siemens MAGNETOM Verio, Philips Achieva and Philips Ingenia. There were 21 patients with EGFR mutation and 17 patients with wild-type EGFR in the whole external testing set. The statistics of the external testing set are shown in Table 2.

**Table 2:** The statistics of the training group and external testing set used in this study

		Training group (n = 155)	External testing set$^a$ (n = 22)	External testing set$^b$ (n = 16)
Age				
Median(range)		57 (46–78)	56 (43-77)	58 (48–81)
Sex				
Male		90 (58.06%)	10 (45.45%)	8 (50.00%)
Female		65 (41.94%)	12 (54.55%)	8 (50.00%)
EGFR Status				
Mutant		83 (53.55%)	12 (54.55%)	9 (56.25%)
Wild		72 (46.45%)	10 (45.45%)	7 (43.75%)

$^a$22 patients diagnosed with LADC BM in Linyi People’s Hospital.

$^b$16 patients diagnosed with LADC BM in Shanghai Chest Hospital

The GTV contours of the BMs were manually outlined by two oncologists (with five and six years of experience, respectively) utilizing MIM Maestro 6.8.2 software. Each oncologist annotated all images, while another oncologist (with 15 years of experience) modified the masks and confirmed the inconsistent areas. The mask for training, validation and testing were transferred to binary images based on the ground truth. To make the proposed network focus on the characteristics of BM regions, the MR images without BM regions were eliminated from the dataset.

### Experimental design

Experiments are designed to demonstrate the effectiveness of the multi-task deep learning model proposed in this study. The ResNet [33], DeSeg [23], UNet3+ [34], radiomics model [14], RN-GAP [35], DenseNet [36], SE-Net [37], UNet [38], RA-UNet [39], Swin-UNet [40], TransUNet [41] deep learning models are set to implement separate single-task training for the classification and segmentation tasks.

### Implementation details

The multi-task loss function is selected as the error measurement index. The adaptive moment estimation (Adam) optimizer [42] is used to train the network, and the initial learning rate is set to 3e-4. The training procedure will be terminated if the validation loss does not improve within 20 epochs. The model is trained on a GeForce-GTX-1080-Ti GPU with 11 GB memory (NVIDIA, Santa Clara, Calif).

### Evaluation metrics

To evaluate the performance of the models, the Dice similarity score (Dice), 95th-percentile Hausdorff distance ($HD_{95}$), precision, and recall metrics are employed to evaluate the BM GTV segmentation results, and the accuracy, recall, precision, and F1-score are employed to evaluate the EGFR genotype prediction results. The Dice score is the most commonly used metric for validating medical image segmentation results, and it can directly show the difference between automatic and ground truth segmentation outputs. $HD_{95}$ is based on calculating the 95th-percentile of the distances between the boundary points in the predicted results and the ground truth. The utilization of the 95th-percentile serves the purpose of mitigating the influence exerted by a small subset of possible outliers. The F1-score, serving as a metric for classification tasks, represents the harmonic mean of recall and precision. For the GTV segmentation evaluation, the precision and recall metrics are calculated in pixels. However, the accuracy, recall, precision, and F1-score are calculated on a per-patient basis for EGFR genotype prediction. The associated functions are shown as follows:

10 $$\begin{aligned} Dice = \frac{{2\left| {X \cap Y} \right| }}{{\left| X \right| + \left| Y \right| }} = \frac{{2 \times TP}}{{2 \times TP + FN + FP}} \end{aligned}$$

where *X* and *Y* represent the ground truth set and the set of predicted results , respectively. *TP*, *FP*, and *FN* denote the numbers of true positives, false positives, and false negatives, respectively.

11 $$\begin{aligned} H{D_{95}} = {K_{95}} \mathop {\max }\limits _{\begin{array}{c} {\scriptstyle {S_A} \in X}\\ {\scriptstyle {S_B} \in Y} \end{array}} \left[ { \min (d(m,{S_A})), \min (d(n,{S_B}))} \right] \end{aligned}$$

where $S_A$ and $S_B$ represent the ground truth of the GTV and the network prediction results , respectively. *X* and *Y* denote the ground truth set and predicted results set. $K_{95}$ indicates the 95th-percentile. $d(m,{S_A})$ is the distance from the surface voxel *m* of the predicted results to the surface of the ground truth. $d(n,{S_B})$ is the distance from the surface voxel *n* of the ground truth to the predicted output results.

12 $$\begin{aligned} Accuracy = \frac{{TP + TN}}{{TP + FP + TN + FN}} \end{aligned}$$

13 $$\begin{aligned} Precision = \frac{{TP}}{{TP + FP}} \end{aligned}$$

14 $$\begin{aligned} Recall = \frac{{TP}}{{TP + FN}} \end{aligned}$$

where TP, FP, and FN denote the number of true positives, false positives, and false negatives, respectively.

15 $$\begin{aligned} F1-score = \frac{{2 \times Precision \times Recall}}{{Precision + Recall}} \end{aligned}$$

### Statistical analysis

**Fig. 5:**
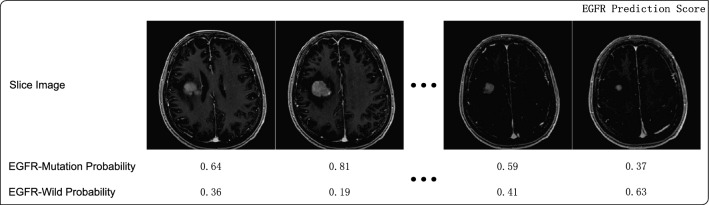
The predicted probabilities of the slice images

The GTV segmentation and EGFR classification results are evaluated on the testing set using the Dice score, $HD_{95}$, accuracy, precision, recall, and F1-score metrics separately. As illustrated in Fig. 5, because the proposed network is a two-dimensional algorithm, each predicted EGFR status pertains to a single MR slice of the corresponding patient. However, certain MR slices, particularly those at the starting or ending slices of the tumor, may contain smaller tumor regions, leading to less tumor information. A post-processing process is introduced to select MR slices with more tumor regions and calculate the mean EGFR mutation probability of these slices. First, for each MR slice, the area of the BM regions output by the GTV segmentation decoder is calculated using the opencv_python (version 4.2.0) software package. Second, the MR slices are arranged in descending order based on their area of the BM region in terms of each patient. Third, for each patient, we select the upper 50% of these slices and compute the arithmetic mean using the predicted EGFR mutation probability of each selected slice. A patient is predicted to have an EGFR mutation status if the mean EGFR mutation probabilities exceeds 0.5, and vice versa.

## Results

### Comparison with the existing algorithms

**Table 3:** The comparison between our proposed model and other algorithms on the internal testing set

Model	GTV segmentation				EGFR genotyping			
	Dice	$HD_{95}$ (mm)	Precision	Recall	Accuracy	Precision	Recall	F1-score
RA-Uent [43]	0.8729 (0.85, 0.90)	3.67	0.9128 (0.90, 0.93)	0.8697 (0.85, 0.89)	–	–	–	–
Swin Unet [40]	0.6395 (0.61, 0.67)	6.51	0.7143 (0.69, 0.74)	0.6574 (0.62, 0.69)	–	–	–	–
TransUnet [41]	0.8565 (0.84, 0.88)	3.78	0.8794 (0.84, 0.89)	0.8631 (0.85, 0.88)	–	–	–	–
Unet [38]	0.8888 (0.86, 0.90)	3.63	0.9031 (0.87, 0.91)	0.9011 (0.89, 0.92)	–	–	–	–
DeSeg [23]	0.8566 (0.83, 0.87)	4.13	0.8890 (0.86, 0.91)	0.8789 (0.85, 0.89)	–	–	–	–
Unet3+ [34]	0.7946 (0.77, 0.82)	4.63	0.7205 (0.70, 0.74)	0.9412 (0.92, 0.96)	–	–	–	–
ResNet-50 [16]	–	–	–	–	0.7879 (0.65, 0.93)	0.8333 (0.71, 0.96)	0.7895 (0.65, 0.93)	0.8108
Radiomics Model [14]	–	–	–	–	0.6061 (0.44, 0.77)	0.6667 (0.51, 0.83)	0.6316 (0.47, 0.80)	0.6486
RN-GAP [35]	–	–	–	–	0.7273 (0.58, 0.88)	0.7778 (0.64, 0.92)	0.7368 (0.59, 0.89)	0.7568
SE-Net [37]	–	–	–	–	0.8182 (0.69, 0.95)	0.8824 (0.77, 0.99)	0.7895 (0.65, 0.93)	0.8333
DenseNet [36]	–	–	–	–	0.7879 (0.65, 0.93)	0.8000 (0.66, 0.94)	0.8421 (0.72, 0.97)	0.8205
MTSA-Net (ours)	0.8914 (0.88, 0.91)	3.58	0.9063 (0.88, 0.92)	0.9035 (0.89, 0.92)	0.8788 (0.77, 0.99)	0.9412 (0.86, 1.00)	0.8421 (0.72, 0.97)	0.8889

The figures enclosed in parentheses indicate the 95% confidence intervals

**Fig. 6:**
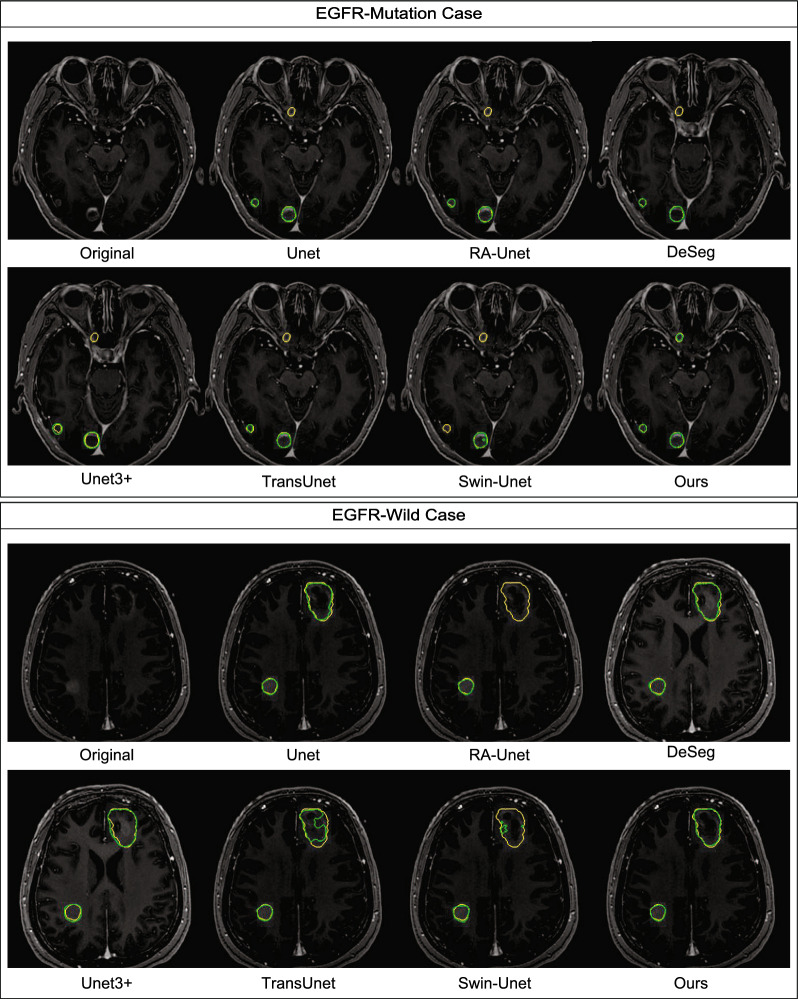
The original slices and GTV segmentation results of two distinct cases. The yellow outline represents the ground truth, and the green outline represents the predicted results

To demonstrate the effectiveness of the proposed network, we compare it with multiple existing single-task methods. The performances of these algorithms are presented in Table 3. Our proposed method achieves remarkable performance in both the EGFR genotyping and GTV segmentation tasks. Specifically, our proposed MTSA-Net achieves an accuracy of 87.88%, a precision of 94.12%, a recall of 84.21% and an F1-score of 0.8889. Compared with single-task classification algorithms such as SE-Net, ResNet, and DenseNet, our proposed method performs better in terms of the four evaluation indices. Significantly, the precision of our proposed model is dramatically improved due to the reduced generation of false EGFR-mutation predictions. The architectures of these single-task classification networks allow them to obtain feature maps that are related to EGFR genotyping from the whole input images, which may capture some unreliable features, especially when the BM area occupies a small proportion of the entire image. In contrast, our proposed MTSA-Net includes a GTV decoding branch, allowing the encoder to derive EGFR genotype-related feature maps from the BM regions to the greatest extent possible. Therefore, our proposed MTSA-Net shows better predictive performance, demonstrating the efficacy of the multi-branch structure and the hybrid CNN-Transformer fusion layer for predicting EGFR mutations.

The experimental results also show that our proposed network outperforms these single-task methods in GTV segmentation tasks. Specifically, our proposed MTSA-Net achieves an average Dice score of 89.14% and an $HD_{95}$ of 3.58 mm. Most of the measurements obtained by our method are the best among all the compared segmentation methods. It is worth mentioning that Swin-UNet is based on a pure transformer architecture as its encoder, which achieves inferior segmentation performance compared with that of the rest of the comparison models. For the characteristics of BM MR images, the U-shaped structure based on convolutional blocks is more suitable. This also illustrates that our proposed MTSA-Net enhances the representations of features by combining convolution operations and Transformer.

As Fig. 6 displays the original 2D slices of two distinct cases and the segmentation results obtained using different methods. The results indicate that one case has the EGFR-mutation subtype, while the other case has the EGFR-wild subtype. In the EGFR-mutation case, three small-size BM lesions are contained in the original slice image. Some GTV delineation results are omitted by all four single-task segmentation algorithms. In the EGFR-wild case, the performance of our proposed model and UNet are quite perfect; their results only require a radiologist to fine-tune the GTV, while the outputs of the other models deviate greatly from the ground truth.

### Model performance achieved on the external testing set

**Fig. 7:**
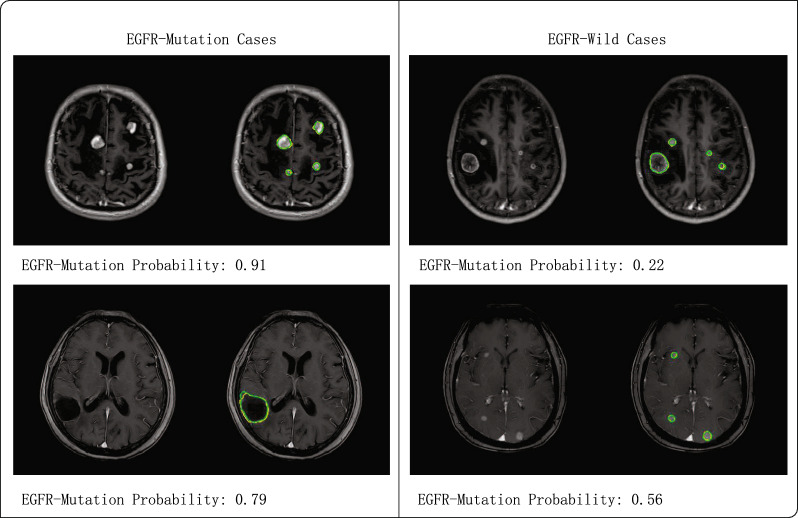
The original slices and prediction results of four distinct patients in the external testing set. The yellow outline represents the BM GTV ground truth, and the green outline represents the predicted BM GTV results. The EGFR-Mutation Probability denotes the mean probability after performing post-processing. A patient is considered to have an EGFR mutation status when the EGFR-Mutation Probability is larger than 0.5, and vice versa

To evaluate the performance and effectiveness of the proposed model, we conduct an assessment using the external testing set obtained from two additional public hospitals. The T1-CE MR images of these cases were acquired using five different MRI scanners to examine the effects of different machines, operators, and other objective factors on model performance. Our proposed method exhibits outstanding performance, achieving an accuracy of 81.58%, a precision of 81.82%, a recall of 85.71% in the EGFR genotyping prediction task and an average Dice score of 85.37% and an $HD_{95}$ of 3.87 mm in the GTV segmentation task. After conducting the comparative analysis, it is found that the variance between the MRI scanner and operator does have some impact on the model’s efficacy. Nevertheless, our proposed model consistently demonstrates favorable performance in terms of robustness. Fig. 7 shows the results output for four distinct patients. The area of the BM region exhibits a positive correlation with the EGFR status predictive capability. A larger BM region provides more lesion information, thereby enhancing the model’s prediction accuracy.

### Ablation experiment

#### Effectiveness of the multi-Task loss function

**Table 4:** Performance of our proposed method using different hyperparameters for the focal loss

Model	GTV segmentation				EGFR genotyping		
	Dice	$HD_{95}$ (mm)	Precision	Recall	Accuracy	Precision	Recall
$\gamma = 2, \alpha = 0.7$	0.8773	3.71	0.8911	0.8932	0.8485	0.8889	0.8421
$\gamma = 2, \alpha = 0.8$	0.8914	3.58	0.9063	0.9035	0.8788	0.9412	0.8421
$\gamma = 2, \alpha = 0.9$	0.8827	3.66	0.9047	0.8922	0.8788	0.8947	0.8947

To demonstrate the effectiveness of the hyperparameters in the focal loss, we compare different combinations. The comparison results are shown in Table 4. Following [30], the preferable approach is to set $\gamma$ to 2 to ensure the ease of the convergence process while increasing the weights of hard samples. $\alpha$ is employed to address the imbalance between positive and negative samples. By definition, the value of $\alpha$ can be set to 0.5 when the numbers of positive and negative examples are approximately equal. When the number of positive examples is lower than the number of negative examples, $\alpha$ can range between 0.5 and 1. After performing parameter sweeping, we set $\gamma$ and $\alpha$ to 2 and 0.8, respectively .

**Table 5:** Performance achieved by our proposed method using different weighting methods

Model	GTV segmentation				EGFR genotyping		
	Dice	$HD_{95}$ (mm)	Precision	Recall	Accuracy	Precision	Recall
Equal weights$^1$	0.8656	3.83	0.9190	0.8626	0.8485	0.8500	0.8947
Ucertain weights (Ours)	0.8914	3.58	0.9063	0.9035	0.8788	0.9412	0.8421

$^1$The equal weights was 0.5: ${L_{\mathrm{{joint}}}} = 0.5{L_{GTV}} + 0.5{L_{EGFR}}$

As shown in Table 5, we confirm the superiority of uncertain weights in the multi-task loss function over certain weights. The results demonstrate that our proposed algorithm, employing uncertain weights, outperforms the network utilizing certain weight combinations in the BM GTV segmentation task. Furthermore, the proposed method also has better sensitivity to EGFR mutations and achieves better accuracy in the EGFR genotyping task. The uncertain weights are helpful for balancing the BM GTV segmentation and EGFR genotyping tasks, thereby preventing any single task from dominating the training process.

## Discussion

In the field of cancer research, an increasing number of machine learning algorithms have been proposed for automated analysis in various clinical application scenarios. A promising area known as radiogenomics has emerged as the state-of-the-art approach in precision medicine; it is a combination of imaging and genomic information implemented using artificial intelligence [44, 45]. Recently, since attention-based neural networks have been demonstrated to be very powerful extractors of shared features for multi-task deep learning models, we are inspired to design a multi-scale attention network to tackle multiple tasks in advanced LADC diagnosis scenarios. This approach presents a potential noninvasive alternative to biopsy procedures for determining the EGFR statuses in advanced LADC cases using brain MR images. Our study introduces a novel multi-task deep learning network called MTSA-Net, which is specifically designed to achieve multi-task collaboration by using features extracted from MR images and emphasizing the connection between GTV and EGFR. This network is designed to predict EGFR mutations for guiding targeted drug treatment plans while automatically and accurately performing GTV segmentation to reduce the intensity of repetitive tasks for radiologists.

We validate the effectiveness of the proposed multi-task deep learning network in terms of improving both the GTV segmentation and EGFR genotyping prediction outcomes. Compared to single-task algorithms, MTSA-Net significantly improves the EGFR genotyping prediction results while achieving better GTV segmentation results. The segmentation task focuses on localizing BM regions, whereas the classification task aims to distinguish EGFR genotypes based on BM imaging features at the volume level. Consequently, the EGFR genotyping classification task is strongly correlated with BM localization and representation, and EGFR genotypes also contribute to the GTV segmentation task. The encoder of the multi-task model underscores its ability to enable shared representations that facilitate accurate BM region localization and significant feature extraction. The MSA block can enhance important information at each scale and adaptively refine tumor boundaries. It can also further improve the specificity of the predicted EGFR statuses by fully exploiting the context information between BMs and the background. We also demonstrate that the proposed multi-task loss function can further improve segmentation and prediction performance of the model by using a learnable weighted matrix.

Our study also has some limitations. First, accurate EGFR genotyping is difficult to achieve in patients with small BMs. From a technical perspective, the main reason for this may be that it is extremely difficult for the encoder to extract effective features from extremely small lesions. Second, the data scale and multi-institution sources remain constrained, making it infeasible to develop a multi-center model based on extensive data. We will continue to expand the dataset and achieve the model’s practical promotion and clinical applicability. Third, this study focuses solely on predicting EGFR mutation statuses, which results in the omission of more specific mutant genotypes and genotypes that exhibit resistance to targeted agents. Future work will be dedicated to predicting more pathological molecules, such as mutations in EGFR exon 19del and 21L858R, and EGFR T790m. Therefore, our future work will not only focus on the design of more advanced algorithms, but also concentrate on collecting a wide range of data to make the model more general.

## Conclusion

In conclusion, we propose MTSA-Net, a multi-task learning network designed for simultaneous EGFR genotype prediction and GTV segmentation. The effectiveness of our proposed framework is assessed on an independent testing set divided by patients’ admission times, and comparative experiments demonstrate its superior performance to that of the existing state-of-the-art methods. Our proposed framework is to be used as a reliable computer-aided system for EGFR genotype prediction and GTV segmentation, enabling the use of a noninvasive method.

## Data Availability

In order to safeguard the confidentiality of the participants, the data pertaining to this study are currently withheld from public access. The data can be shared upon request.

## Code availability

The code can be shared upon request.
